# Gadoxetic acid-enhanced MRI in hepatocellular carcinoma: a comprehensive review of diagnostic, surveillance, and treatment response prediction and assessment

**DOI:** 10.1007/s11604-025-01870-x

**Published:** 2025-09-15

**Authors:** Kumi Ozaki, Yukichi Tanahashi, Satoshi Goshima

**Affiliations:** Department of Radiology, Hamamatsu University School of Medicine, 1-20-1, Handayama, Chuo-ku, Hamamatsu City, Shizuoka 431-3192 Japan

**Keywords:** Gadoxetic acid, Magnetic resonance imaging, Hepatocellular carcinoma, Hepatobiliary phase, Liver Imaging Reporting and Data System

## Abstract

Gadoxetic acid-enhanced magnetic resonance imaging (MRI) has become a pivotal imaging modality in hepatocellular carcinoma (HCC) management, offering unique advantages owing to its hepatocyte-specific contrast properties. Its technical foundation includes optimized dynamic phase imaging and hepatobiliary phase (HBP) acquisition, which together provide functional information unattainable with conventional extracellular contrast agents. This modality enhances sensitivity in detecting HCC and enables superior characterization of focal liver lesions based on hepatocyte-specific uptake patterns. In high-risk patients with chronic liver disease, gadoxetic acid-enhanced MRI facilitates the early detection of small and early-stage HCCs, enabling timely intervention and potentially improving clinical outcomes. Beyond diagnosis, gadoxetic acid-enhanced MRI aids in predicting treatment response by evaluating tumor biological characteristics. Key imaging biomarkers include: hyperintense or heterogeneous HCC on HBP, suggesting tumor immune microenvironment; peritumoral hypointensity on HBP, suggesting microvascular invasion; and clear hypointensity on HBP with several other findings, indicating vessels encapsulating tumor clusters, characteristic of the macrotrabecular-massive HCC subtype. These biomarkers support a comprehensive evaluation of histological differentiation and biological aggressiveness. Furthermore, this modality demonstrates superior accuracy in assessing local therapy effectiveness and monitoring systemic treatment responses compared to conventional imaging. Major international hepatology societies have incorporated gadoxetic acid-enhanced MRI into their HCC diagnostic algorithms, albeit with regional differences in emphasis. Eastern guidelines (e.g., from the Japan Society of Hepatology and the Asian Pacific Association for the Study of the Liver) prioritize sensitivity, whereas Western guidelines (e.g., from the European Association for the Study of the Liver and the Liver Imaging Reporting and Data System) emphasize specificity. Despite certain limitations, including potential suboptimal arterial phase visualization, challenges in interpreting the transitional phase, higher cost, and longer examination times, gadoxetic acid-enhanced MRI remains an indispensable tool in precision oncology, enabling personalized treatment strategies and supporting optimal patient outcomes through comprehensive HCC characterization and accurate treatment monitoring.

## Introduction

Hepatocellular carcinoma (HCC) is the most prevalent type of primary liver cancer and the third leading cause of cancer-related mortality worldwide, with its incidence continuing to rise globally [1]. The development of HCC is strongly associated with underlying chronic liver diseases, particularly cirrhosis, which constitutes a major predisposing factor. While historically associated with viral hepatitis, the epidemiology of HCC has evolved to include alcohol-related liver disease and metabolic dysfunction-associated steatotic liver disease as increasingly prominent etiologies [1, 2]. Early detection of HCC is critical, as this enables access to curative treatment modalities such as surgical resection, liver transplantation, or local ablation, all of which have been shown to significantly improve overall survival [3].

Noninvasive diagnosis relies on several imaging modalities, including dynamic ultrasonography (US), computed tomography (CT), contrast-enhanced magnetic resonance imaging (MRI) using extracellular contrast agents, and MRI with hepatocyte-specific contrast agents. Among these, MRI using hepatocyte-specific contrast agents is particularly valuable owing to its high specificity for HCC detection and characterization [4, 5].

Gadoxetic acid (gadolinium ethoxybenzyl diethylenetriamine pentaacetic acid; Eovist or Primovist, Bayer Healthcare, Berlin, Germany) revolutionized liver imaging as one of the hepatocyte-specific contrast agents. Although gadobenate dimeglumine (i.e., Gd-BOPTA, Multihance, Bracco, Milan, Italy) was clinically available earlier, particularly in Europe, gadoxetic acid has become a pivotal agent in hepatocyte-specific liver imaging. Following intravenous administration, gadoxetic acid with sufficient hepatobiliary excretion permits hepatobiliary phase (HBP) imaging in addition to conventional dynamic contrast phases [6]. This dual pharmacokinetic profile enables simultaneous assessment of vascular perfusion characteristics and hepatocellular functional status within a single examination. This integrated approach significantly enhances comprehensive HCC management and is now a cornerstone of modern diagnostic protocols.

This review explores the multifaceted clinical applications of gadoxetic acid-enhanced MRI in HCC management, including its role in improving diagnostic accuracy, guiding surveillance strategies, and predicting and monitoring treatment response, as well as highlighting current limitations and pitfalls.

## Pharmacokinetics and distinctive imaging characteristics of gadoxetic acid

### Biphasic enhancement pattern: dynamic and hepatobiliary phases

**Fig. 1:**
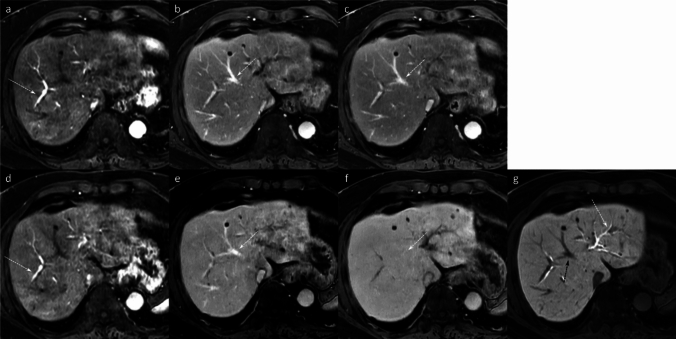
Comparison of the dynamic contrast-enhanced MRI using two different contrast agents. A 73-year-old man with pancreatic adenocarcinoma (not shown) underwent two MRI examinations within 1 week, each utilizing a different contrast agent. The first MRI was performed using an extracellular contrast agent (ECA) (**a**–**c**), and the second using gadoxetic acid (**d**–**g**). In the late hepatic arterial phase, both ECA (**a**) and gadoxetic acid (**d**) demonstrate marked arterial enhancement and early portal vein opacification (arrows). Although gadoxetic acid uptake in the hepatic parenchyma may begin as early as the portal venous phase, the appearance of the portal venous phase with ECA (**b**) and gadoxetic acid (**e**) is similar, with the middle hepatic veins (arrows) being hyperintense relative to the background parenchyma. At approximately 3 min post-injection, the delayed phase with ECA (**c**) and transitional phase with gadoxetic acid (**f**) are acquired. During this phase, gadoxetic acid distributes into both the extracellular and intracellular spaces, rendering the middle hepatic vein (arrow) isointense to the background parenchyma. In contrast, the middle hepatic vein (arrow) remains hyperintense to the background parenchyma on the delayed phase with ECA (c). In the hepatobiliary phase (**g**), gadoxetic acid is predominantly cleared from the extracellular space and accumulates mainly in the intracellular space and bile ducts. Consequently, the liver parenchyma appears more enhanced than the intrahepatic vessels (black arrow), and gadoxetic acid is visualized within the bile ducts (white arrow)

Gadoxetic acid-enhanced MRI enables the comprehensive assessment of liver lesions through its distinct biphasic enhancement pattern. Following intravenous administration, gadoxetic acid initially behaves similarly to extracellular contrast agents, providing valuable perfusion information essential for characterizing lesion enhancement patterns during the dynamic phases (arterial, portal venous, and transitional) (Fig. 1) [6]. Subsequently, the hepatobiliary phase (HBP), typically acquired 15–20 min post-injection, offers a distinct advantage by reflecting the unique hepatocyte-specific uptake of the agent [7].

### Mechanism of hepatocyte uptake and biliary excretion leading to hepatobiliary phase

Gadoxetic acid undergoes a complex hepatocellular uptake and biliary excretion process. After circulating through the vascular compartment similar to conventional extracellular contrast agents during the dynamic phases, gadoxetic acid is selectively taken up from the sinusoidal blood by functioning hepatocytes via organic anion transporting polypeptide (OATP)1B3 on the basolateral membrane. Once internalized, it is transported across the hepatocyte and actively excreted into bile canaliculi through multidrug resistance-associated protein 2 (MRP2) on the apical membrane. This hepatocyte-specific uptake and biliary excretion mechanism results in progressive liver parenchymal enhancement, while non-hepatocytic lesions remain hypointense, creating the characteristic high lesion-to-liver contrast observed in the HBP [6]. This high-contrast imaging not only enhances lesion detection sensitivity but also enables precise lesion characterization based on the distinct signal intensity patterns observed during HBP.

### Role in HCC diagnosis

#### Typical HCC imaging findings

**Fig. 2:**
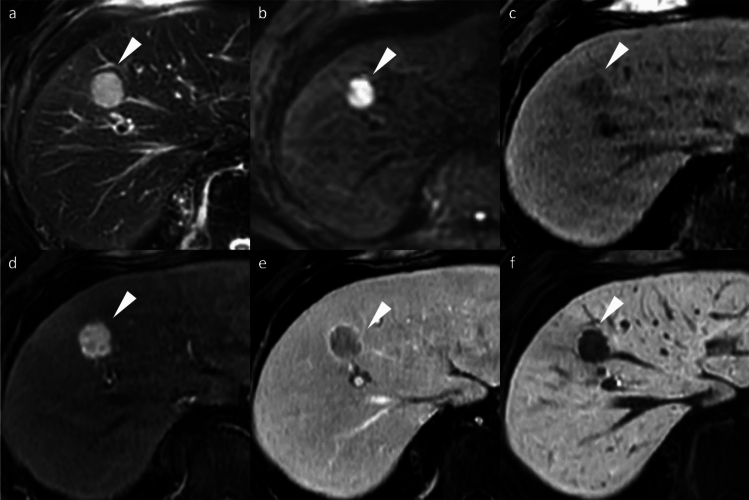
Hepatocellular carcinoma in a 79-year-old man, showing typical imaging findings. A nodule (arrowheads) located in segment 4 appears clearly hyperintense on fat-suppressed T2-weighted images (**a**) and demonstrates diffusion restriction on diffusion-weighted images (**b**). The nodule displays hypointensity on pre-contrast image (**c**), non-rim arterial phase hyperenhancement (**d**) with portal venous washout (**e**), and clear hypointensity on the hepatobiliary phase (**f**). The nodule was pathologically proven to be hepatocellular carcinoma following surgical resection

Typical HCC demonstrates non-rim arterial phase hyperenhancement (APHE), portal venous washout, and HBP hypointensity. HBP hypointensity, observed in 85–95% of HCCs, reflects impaired hepatocyte function and reduced gadoxetic acid uptake (Fig. 2). Together, these findings constitute the characteristic imaging signature of HCC on gadoxetic acid-enhanced MRI [8]. The high specificity of these features is incorporated into the Liver Imaging Reporting and Data System (LI-RADS) framework as major diagnostic features [8].

**Fig. 3:**
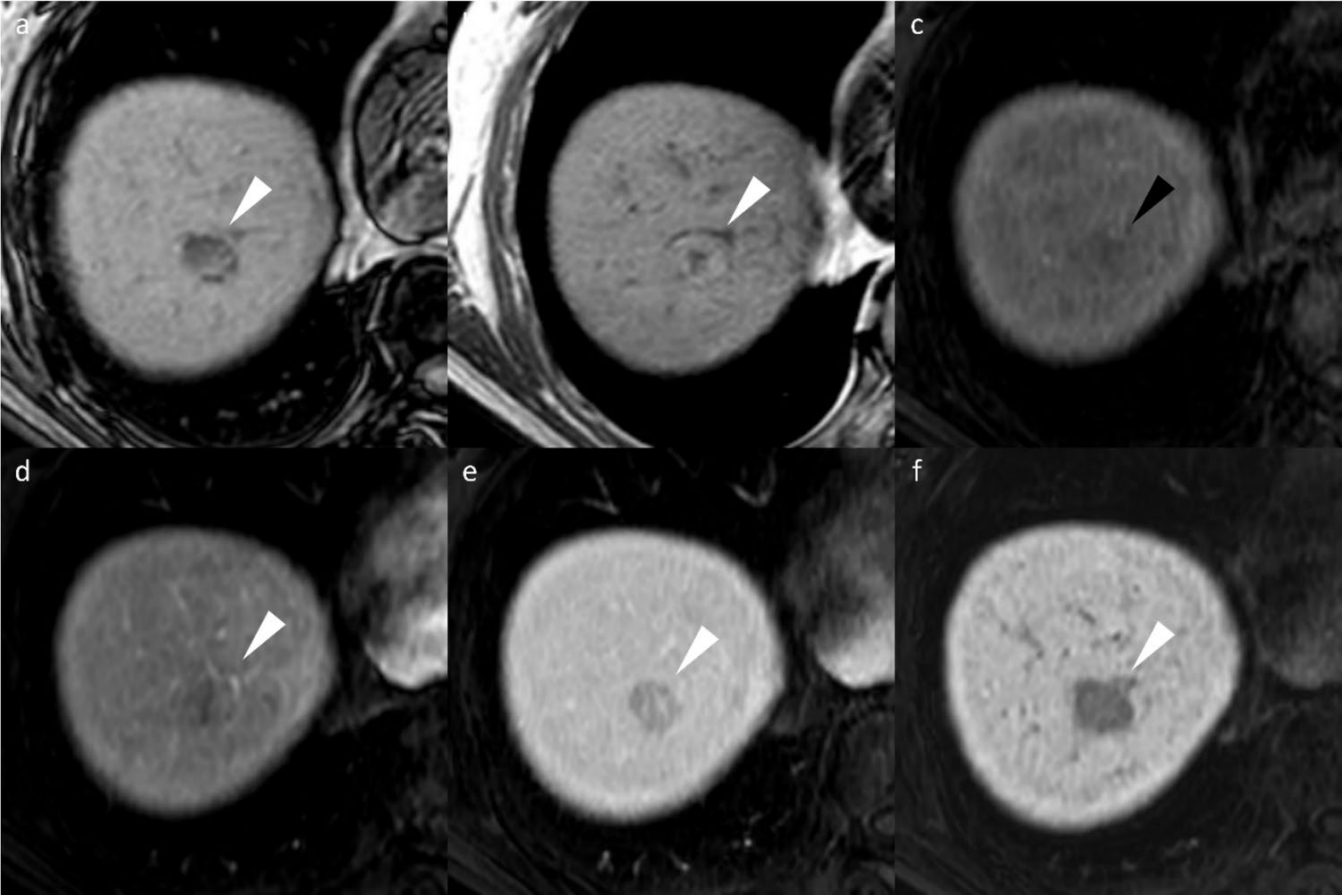
Fat-containing hepatocellular carcinoma in a 74-year-old man. Opposed-phase (**a**) and in-phase (**b**) images depicting signal loss within the nodule (arrowheads). The nodule (arrowheads) appears isointense relative to the background liver parenchyma on the pre-contrast image (**c**) and does not exhibit non-rim arterial phase hyperenhancement on the arterial phase image (**d**). It shows hypointensity on the transitional phase image (**e**) and clear hypointensity on the hepatobiliary phase image (**f**). Histopathological examination following surgical resection confirmed the diagnosis of hepatocellular carcinoma

**Fig. 4:**
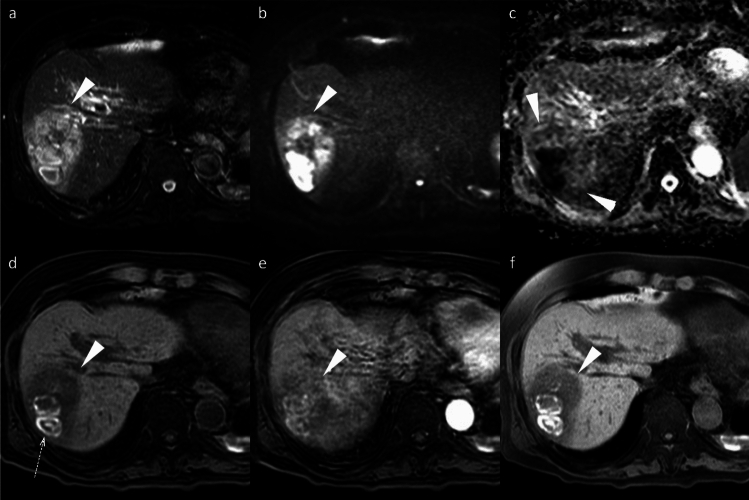
Hepatocellular carcinoma in a 73-year-old man. The mass (arrowheads) in segment 8 displays inhomogeneous hyperintensity on fat-suppressed T2-weighted imaging (**a**) and inhomogeneous diffusion restriction on diffusion-weighted imaging (**b**). A part of the mass shows a reduced apparent diffusion coefficient value compared to the background liver parenchyma (**c**). Pre-contrast image reveals intralesional blood products (arrow) (**d**). The arterial phase image is compromised by transient severe motion, making reliable evaluation of non-rim arterial phase hyperenhancement difficult (**e**). On the hepatobiliary phase, the lesion appears hypointense, except for areas corresponding to hemorrhage (**f**). Histopathological analysis following biopsy confirmed the diagnosis of hepatocellular carcinoma

**Fig. 5:**
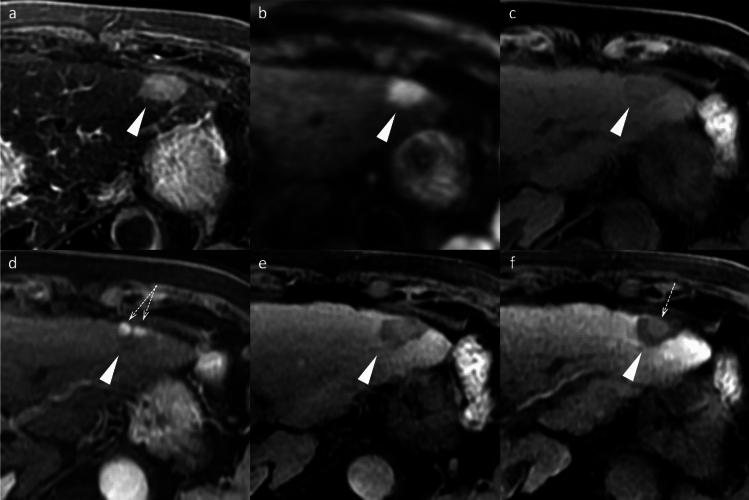
Hepatocellular carcinoma with a nodule-in-nodule appearance in an 82-year-old woman. A nodule (arrowheads) in segment 3 displays homogeneous hyperintensity on fat-suppressed T2-weighted imaging (**a**) and homogeneous diffusion restriction on diffusion-weighted imaging (**b**). The nodule appears hypointense relative to the background liver on the pre-contrast image (**c**). In the arterial phase (**d**), a portion of the nodule exhibits hyperenhancement, consistent with a nodule-in-nodule appearance (arrows). The nodule appears hypoenhancing relative to the background liver on the transitional phase image (**e**), and the nodule-in-nodule appearance is again evident on the hepatobiliary phase image (arrow) (**f**)

Beyond the major features, ancillary findings such as the presence of a non-enhancing capsule, fat in mass (Fig. 3), mosaic architecture (Fig. 4), nodule-in-nodule (Fig. 5), and blood products in mass (Fig. 4) strongly support HCC diagnosis [8]. Other supportive findings include subthreshold growth, diffusion restriction (Figs. 2 and 4), mild-to-moderate T2 hyperintensity (Figs. 2, 4, and 5), fat or iron sparing, and transitional phase or HBP hypointensity (Figs. 2, 3, 4, 5) [8]. These features are particularly valuable when lesions do not exhibit the typical vascular profile of HCC. Thus, the diagnostic utility of gadoxetic acid-enhanced MRI lies not solely in the hepatobiliary phase but in the comprehensive evaluation across dynamic contrast-enhanced phases and supplementary sequences such as diffusion-weighted imaging.

#### Differentiation from other hepatocellular lesions

##### Early HCC and high-grade dysplastic nodules (HGDN)

**Fig. 6:**
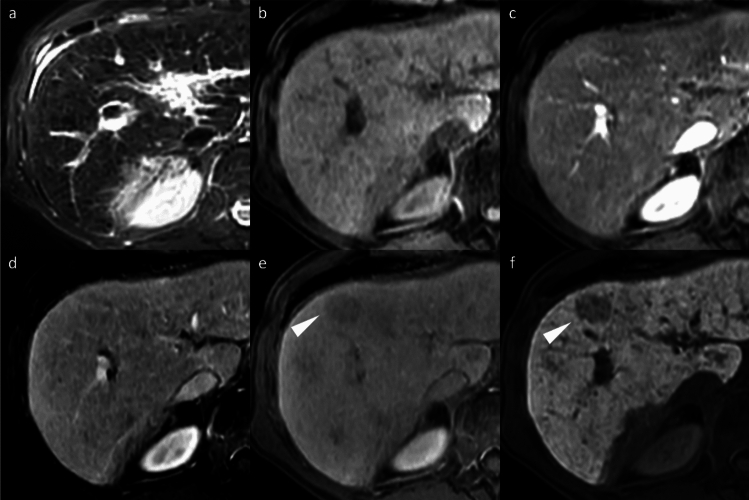
Hepatobiliary phase hypointense nodule without arterial phase hyperenhancement in a 69-year-old man with hepatitis B virus-related cirrhosis. A nodule (arrowhead) appears hypointense relative to the surrounding liver on the hepatobiliary phase image (**f**). It shows mild hypointensity on the transitional phase image (**e**; arrowhead), but no arterial phase hyperenhancement (**c**) or portal venous washout (**d**). The lesion is inconspicuous on fat-suppressed T2-weighted (**a**) and pre-contrast T1-weighted images (**b**)

HBP hypointense nodules without APHE are a unique finding on gadoxetic acid-enhanced MRI. Pathologic assessment reveals that approximately 74% of such nodules represent early HCCs, while 10% are dysplastic nodules (Fig. 6) [9].

Hepatocarcinogenesis progresses in a multistep sequence: from low-grade dysplastic nodule (LGDN) to HGDN, early HCC, well-differentiated HCC, nodule-in-nodule HCC, and ultimately, moderately differentiated HCC. Early HCC represents the earliest morphologically recognizable stage, characterized by subtle architectural changes and minimal cytological atypia, while hepatocellular function is relatively preserved. On gadoxetic acid-enhanced MRI, early HCCs typically appear hypointense during the HBP due to decreased expression of OATP1B3, which mediates gadoxetic acid uptake [9, 10].

Unlike progressed HCCs, which demonstrate characteristic non-rim APHE due to neoangiogenesis and arterial supply predominance, early HCC often lacks APHE, reflecting the minimal or no vascular changes in the early stages of hepatocarcinogenesis [9].

HGDNs typically exhibit iso- or hypointensity on HBP imaging and generally do not show APHE, although mild hyperintensity may be seen in cases with preserved hepatocyte function [9].

Importantly, of these HBP hypointense nodules without APHE, approximately 8% are LGDNs or regenerative nodules that do not require aggressive treatment [11]. While elevated α-fetoprotein levels and certain MRI features, including well-defined margin (odds ratio [OR], 5.5), hypointensity on pre-contrast T1-weighted imaging (OR, 3.2), intermediate hyperintensity on T2-weighted imaging (OR, 3.4), and restricted diffusion (OR, 1.9), may help differentiate advanced HCC from LGDNs or regenerative nodules, careful interpretation is required [11]. LI-RADS considers APHE as an important finding that enhances the specificity of HCC diagnosis [8].

##### Focal nodular hyperplasia (FNH) and FNH-like lesion

**Fig. 7:**
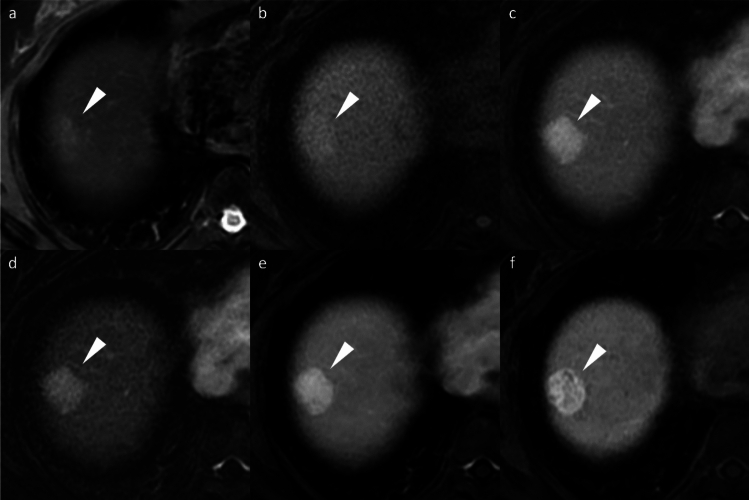
Focal nodular hyperplasia in a 47-year-old woman. A nodule (arrowheads) in segment 8 shows mild hyperintensity on the fat-suppressed T2-weighted image (**a**) and the pre-contrast image (**b**). The nodule displays non-rim arterial phase hyperenhancement (**c**) without portal venous washout (**d**), and prolonged hyperenhancement in the transitional phase (**e**). On the hepatobiliary phase image, the nodule appears distinctly hyperintense relative to the background liver (**f**). Typical imaging findings led to the diagnosis of focal nodular hyperplasia

Differentiating gadoxetic acid-enhanced hyperintense HCCs from FNH can be challenging owing to overlapping imaging features, as both may exhibit non-rim APHE (Fig. 7) [12]. Key differentiating features that favor hyperintense HCC include a lower apparent diffusion coefficient (ADC), portal venous phase washout pattern, the presence of a capsule-like rim, and a mosaic appearance [8]. Conversely, a central scar is characteristic of FNH [12].

**Fig. 8:**
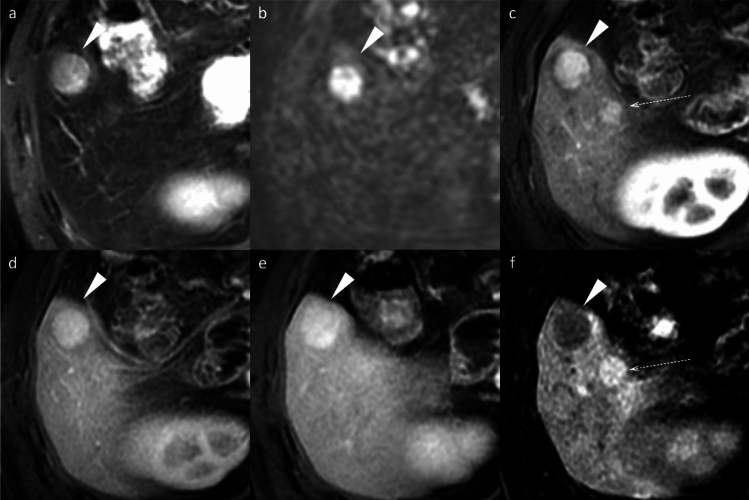
Focal nodular hyperplasia (FNH)-like lesions in a 23-year-old man with alcoholic liver disease. Multiple FNH-like lesions were identified in a 23-year-old man with alcoholic liver disease. A nodule in segment 5 displayed hyperintensity on fat-suppressed T2- and diffusion-weighted images (**a**, **b**; arrowheads). The nodule demonstrated non-rim arterial phase hyperenhancement (**c**; arrowhead) without washout on the portal venous phase (**d**) and prolonged enhancement in the transitional phase (**e**; arrowheads). In the hepatobiliary phase, the nodule appeared hypointense relative to the background liver (**f**; arrowhead). No central scar was observed. The nodule was pathologically confirmed to be an FNH-like lesion on biopsy. In contrast, other nodules showed typical hyperintensity in the hepatobiliary phase compared to the background liver (**f**; arrow)

FNH-like lesions occur in highly diverse pathological settings and present additional diagnostic complexity, particularly in cirrhotic livers, as they may mimic HCC on imaging. Up to 30% of FNH-like lesions in patients with cirrhosis demonstrate HBP hypointensity (Fig. 8) [13], and in one study, three of nine (33%) FNH-like lesions would have been misdiagnosed as HCC using standard diagnostic criteria [14]. Independent predictors of FNH-like lesions include small size (< 1.6 cm), iso- or hypointensity on diffusion-weighted MRI, and absence of portal venous washout. If all three criteria are met, then a 100% specificity for FNH-like lesions can be achieved [13].

In addition, FNH-like lesions specifically occurring in alcoholic cirrhosis demonstrate key differentiating imaging features on imaging, including hypervascularity despite small size, hyperintensity on T1-weighted images, no signal drop on chemical shift images, and incomplete gadoxetic acid uptake on hepatobiliary phase [15].

##### Hepatocellular adenomas (HCAs)

Differentiating HCA from HCC presents diagnostic challenges due to overlapping imaging features, particularly non-rim APHE. However, HCC typically exhibits washout during the portal venous phase [8], whereas HCA generally shows persistent enhancement [16]. HCA imaging features vary by molecular subtype, and key clinical differentiating features include occurrence in non-cirrhotic livers and younger patients with oral contraceptive history.

**Fig. 9:**
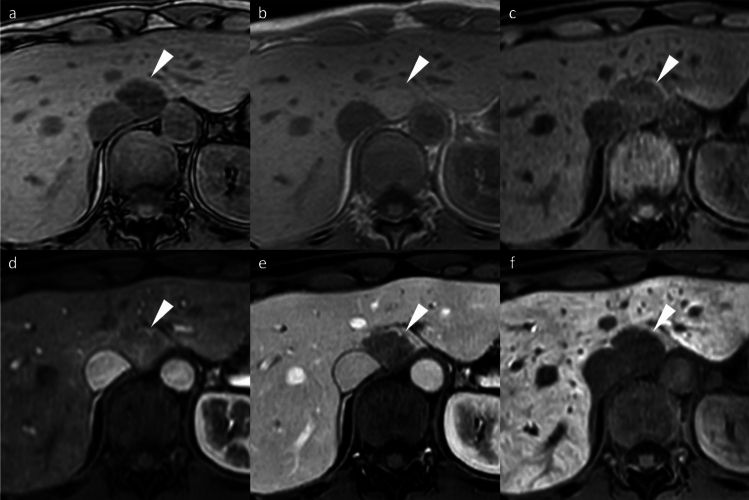
Hepatocyte nuclear factor 1α (HNF-1α)-inactivated hepatocellular adenoma in a 27-year-old woman. Opposed-phase (**a**) and in-phase (**b**) images depict the presence of intralesional fat (arrowheads). The nodule (arrowheads) appears hypointense relative to the background liver parenchyma on the pre-contrast image (**c**), shows mild arterial phase hyperenhancement (**d**) with portal venous washout (**e**), and clear hypointensity on the hepatobiliary phase image (**f**). Histopathological analysis following biopsy confirmed the diagnosis of HNF-1α-inactivated hepatocellular adenoma

HNF1α-inactivated HCA (H-HCA) demonstrates signal dropout on chemical shift imaging due to intralesional fat, its most distinctive feature. H-HCAs exhibit non-rim APHE but become hypoenhancing during the portal venous phase, mimicking washout, and are markedly hypointense on HBP (Fig. 9). The characteristically mild arterial enhancement can aid differential diagnosis [16].

**Fig. 10:**
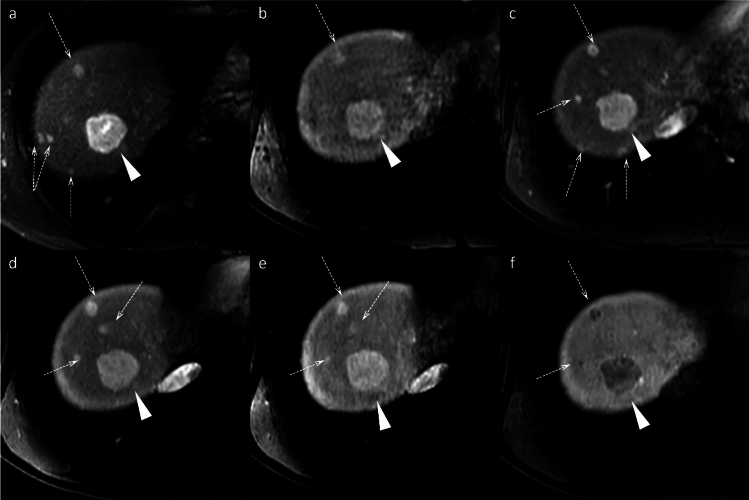
Inflammatory hepatocellular adenomas in a 21-year-old man with glycogen storage disease type I. A mass (arrowheads) located in segment 8 shows clear hyperintensity on fat-suppressed T2-weighted image, with an atoll sign evident (**a**). The mass displays hyperintensity on pre-contrast image (**b**), non-rim arterial phase hyperenhancement (**c**) without portal venous washout (**d**), and hyperenhancement on the transitional phase image (**e**). The mass shows homogeneous hypointensity on the hepatobiliary phase image (**f**). Multiple additional small nodules (arrows), suspicious for hepatocellular adenoma, are also noted throughout images (**a**–**f**)

I-HCAs typically exhibit persistent enhancement rather than washout. The “atoll sign” occurs in only 30% but demonstrates high specificity when present (Fig. 10) [16].

**Fig. 11:**
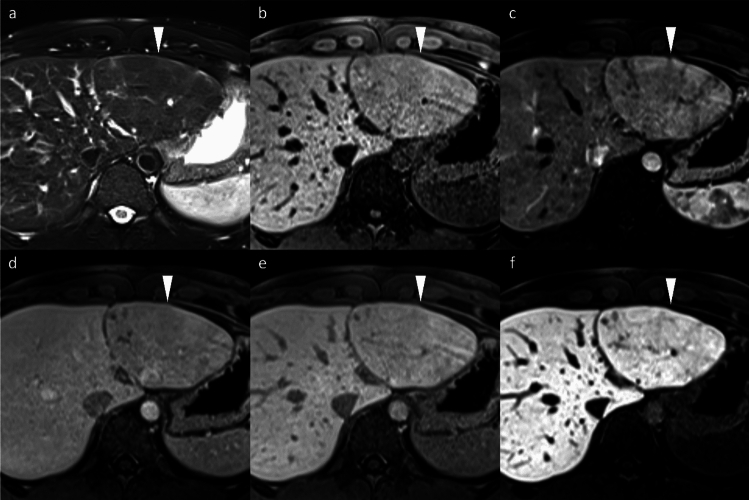
β-Catenin-mutated hepatocellular adenoma in a 23-year-old man. A mass (arrowheads) located in the lateral segment demonstrates an isointense signal on both the fat-suppressed T2-weighted image (**a**) and the pre-contrast image (**b**). The mass displays non-rim arterial phase hyperenhancement (**c**) without portal venous washout (**d**) and hypointensity on the transitional phase image (**e**). The mass shows homogeneous hyperintensity on the hepatobiliary phase image (**f**). The nodule was pathologically proven to be β-catenin-mutated hepatocellular adenoma following surgical resection. Imaging characteristics do not reliably differentiate this lesion from hepatocellular carcinoma; however, the absence of portal venous washout may serve as a useful distinguishing feature

β-Catenin-mutated HCA (β-HCA) presents significant diagnostic challenges due to imaging similarities with HCC, exhibiting non-rim APHE and HBP hyperintensity. The absence of washout serves as a key differentiating feature (Fig. 11). β-HCAs demonstrate substantially higher malignant potential, with exon 3-mutated β-HCAs showing a 20–40% transformation rate [16]. Exon 3 mutations show strong, diffuse glutamine synthetase (GS) expression, while other mutations show patchy staining [17]. Theoretically, exon 3-mutated HCAs should show distinct HBP hyperintensity, but this relationship requires further investigation [16].

#### Differentiation from other liver tumors

##### Intrahepatic cholangiocarcinoma (iCCA)

**Fig. 12:**
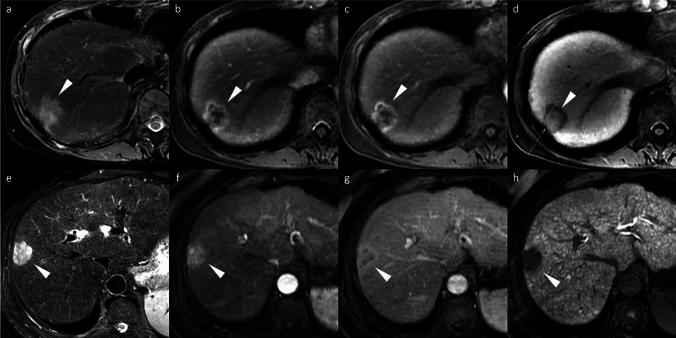
Two types of mass-forming intrahepatic cholangiocarcinoma (ICC) in a 63-year-old woman (**a**–**d**) and a 69-year-old man with hepatitis C virus-related cirrhosis (**e**–**h**). In the 63-year-old woman, ICC at the posterior segment (arrowheads) shows hyperintensity on fat-suppressed T2-weighted imaging (**a**), target appearance in the arterial phase (**b**), and portal venous phase (**c**), as well as intermediate signal intensity or target appearance (arrow) in the hepatobiliary phase (**d**), a pattern referred to as “cloud enhancement.” In the 69-year-old man, ICC at the anterior segment (arrowheads) shows hyperintensity on fat-suppressed T2-weighted imaging (**e**), arterial phase hyperenhancement (**f**) with portal venous washout (**g**), and hypointensity in the hepatobiliary phase (**h**). Differentiation from hepatocellular carcinoma is challenging based on imaging features

Non-HCC malignancies, including iCCA, frequently exhibit a targetoid imaging appearance, characterized by rim APHE, Peripheral “washout,” Delayed central enhancement, targetoid restriction on diffusion-weighted image, and targetoid transitional phase or HBP (Fig. 12) [7]. These targetoid imaging appearances are a key imaging feature for distinguishing iCCA from HCC on gadoxetic acid-enhanced MRI.

The targetoid appearance in the HBP, also referred to as “cloud enhancement,” is attributed to contrast retention within the expanded extracellular volume caused by fibrous stroma, similar to that observed with conventional extracellular contrast agents [18]. However, diagnostic challenges arise because atypical HCCs with prominent fibrosis or central scars can mimic iCCA, and “small duct type” iCCAs can exhibit non-rim APHE and diffusion restriction, resembling HCC (Fig. 12) [19].

##### Liver metastasis

**Fig. 13:**
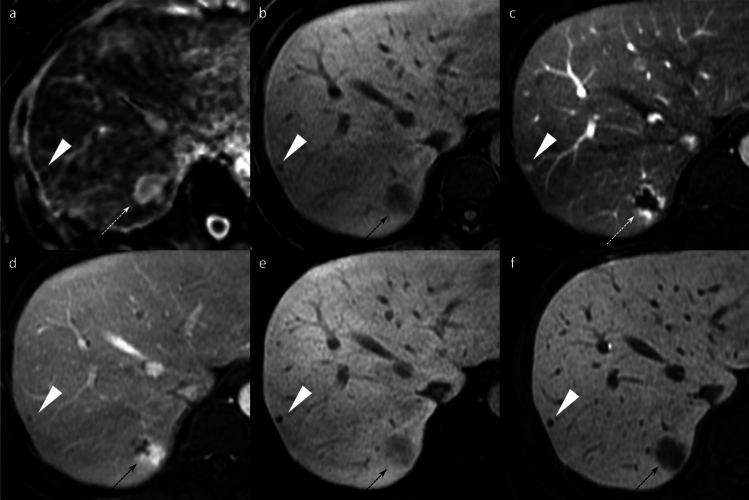
Liver metastasis and cavernous hemangioma in a 60-year-old woman with a history of pancreatic neuroendocrine neoplasm (G2). A small hypervascular liver metastasis (arrowheads) displays lower apparent diffusion coefficient values compared to the background liver (**a**). Hypervascular liver metastasis (arrowheads) displays slight hypointensity on the pre-contrast image (**b**), non-rim arterial phase hyperenhancement (**c**) with portal venous washout (**d**), hypointensity in the transitional phase (**e**), and clear hypointensity in the hepatobiliary phase (**f**). In contrast, a cavernous hemangioma (arrows) displays higher apparent diffusion coefficient values compared to the background liver (**a**). Hemangioma (arrow) displays hypointensity on the pre-contrast image (**b**), peripheral nodular enhancement during the arterial phase (**c**), and progressive centripetal fill-in in the portal venous phase (**d**). In the transitional phase (**e**), hemangioma exhibits pseudo-washout and appears distinctly hypointense in the hepatobiliary phase (**f**)

Hypovascular liver metastases show similar low signal intensity on HBP imaging; however, they typically exhibit rim APHE, allowing for differentiation [19]. Nevertheless, metastases from hypervascular primary tumors, such as neuroendocrine neoplasms or renal cell carcinoma, often demonstrate non-rim APHE with portal venous washout and HBP hypointensity, making differentiation challenging (Fig. 13) [20]. The existence or previous history of primary malignancy represents the most important diagnostic consideration.

### Role in HCC surveillance

#### Comparison of gadoxetic acid-enhanced MRI with ultrasound and CT in surveillance programs

The primary objective of HCC surveillance programs is early detection—ideally at very early or early stages—when curative treatments are most feasible and effective. Biannual US remains the standard surveillance method owing to its accessibility, safety, and low cost. However, US frequently suffers from low sensitivity and a high rate of technically inadequate examinations, limiting its effectiveness [4, 5, 21].

Gadoxetic acid-enhanced MRI demonstrates superior diagnostic performance compared to conventional modalities, including US and contrast-enhanced CT or MRI with extracellular contrast agents. While contrast-enhanced US achieved 84% sensitivity, gadoxetic acid-enhanced MRI provided the highest pooled per-lesion sensitivity (86%) and positive predictive value (94%) [22]. This advantage is particularly pronounced for lesions < 30 mm, where gadoxetic acid-enhanced MRI shows significantly higher sensitivity than contrast-enhanced US while maintaining comparable specificity [23].

In high-risk populations, surveillance with gadoxetic acid-enhanced MRI yielded a higher HCC detection rate (86.0%) and lower false-positive findings (3.0%) compared to US (27.9% and 5.6%, respectively) [4]. Similarly, meta-analyses demonstrate superior performance over contrast-enhanced CT, with a pooled sensitivity of 0.91 for gadoxetic acid-enhanced MRI using 3 T scanners versus 0.74 for CT [24]. MRI also offers enhanced tissue contrast and can improve early-stage HCC staging when added to CT examination [4]. However, in current clinical practice, despite its superior diagnostic performance, the use of gadoxetic acid-enhanced MRI for routine surveillance is limited in most clinical settings due to high cost, limited scanner availability, and poor examination throughput compared with ultrasound. Therefore, gadoxetic acid-enhanced MRI is generally applied for problem-solving or in high-risk subgroups rather than being routinely adopted for population-based surveillance programs.

#### Detection of small HCC with gadoxetic acid-enhanced MRI

HBP imaging represents a major advantage of gadoxetic acid-enhanced MRI. By providing high signal-to-noise and contrast-to-noise ratios, HBP imaging enhances lesion conspicuity, particularly for small lesions, more effectively than other imaging sequences [25]. For tumors ≤ 2 cm, gadoxetic acid-enhanced MRI demonstrated significantly higher sensitivity than CT (87% vs. 73%; *p* = 0.02) and a lower negative likelihood ratio (0.13 vs. 0.28; *p* = 0.01). In contrast, extracellular contrast-enhanced MRI showed mixed results depending on lesion size, achieving significantly higher sensitivity than CT for lesions > 2 cm (88% vs 79%) and < 1 cm (69% vs 48%), but no significant differences for 1–2 cm lesions. These findings highlight the robust diagnostic capability of gadoxetate-enhanced MRI in detecting small HCC lesions, with a 14% improvement in sensitivity and a 54% reduction in negative likelihood ratio compared to CT [26].

In liver transplantation candidates, the inclusion of HBP images has been reported to significantly improve the diagnostic accuracy of gadoxetic acid-enhanced liver MRI for detecting 1–2 cm HCC lesions [27].

#### Impact on early-stage HCC detection and patient outcomes

The superior sensitivity of gadoxetic acid-enhanced MRI, compared to US, contrast-enhanced CT, and MRI with extracellular contrast agents, is primarily attributed to its ability to detect early HCC during HBP [4, 28]. Early-stage HCC is typically identifiable only on HBP and lacks non-rim APHE. Histopathologically, these nodules often correspond to either early HCC or high-grade dysplastic nodules [9], both of which carry a significant risk of developing hypervascularity and progressing to typical HCC [9]. Therefore, these nodules require careful risk stratification and close surveillance for optimal clinical management.

HBP hypointense nodules without APHE have a reported prevalence of 12.6% in high-risk populations [29]. Primary risk factors for malignant transformation include: nodule size ≥ 10 mm (hazard ratio [HR]: 2.95; 95% confidence interval [CI]: 1.94–4.20) [30], T2 hyperintensity (HR: 4.21; 95% CI: 1.15–15.40) [30], diffusion restriction on diffusion-weighted imaging (HR: 5.83; 95% CI: 1.42–23.95) [31], and previous HCC history [31].

These HBP hypointense, non-APHE nodules are independent risk factors for intrahepatic distant recurrence in HCC patients, regardless of treatment modality [30]. A meta-analysis reported a pooled HR of 2.44 (95% CI: 1.99–2.98), with a higher recurrence risk following radiofrequency ablation (HR: 3.07) compared to surgical resection (HR: 2.14), without significant heterogeneity across studies [32].

#### Cost-effectiveness and practical considerations for widespread implementation

Although full-sequence MRI, including gadoxetic acid-enhanced MRI, demonstrates high sensitivity for HCC surveillance, its widespread implementation is limited owing to its high cost, restricted availability, and lengthy scan times. However, economic analyses suggest that gadoxetic acid-enhanced MRI, when performed after CT, can be a cost-effective strategy for detecting additional HCCs in patients with early-stage disease who are candidates for curative treatment (excluding liver transplantation) [33, 34]. To address the challenge of lengthy scan times and improve feasibility for broader surveillance, recent advances in MRI technology and deep learning-based image reconstruction have enabled the acquisition of higher-quality images in shorter scan times [35–41]. In addition, the utility of abbreviated MRI protocols is under active investigation [42].

### Treatment response prediction

Gadoxetic acid-enhanced MRI provides valuable insights into the biological characteristics of HCC before treatment initiation, primarily through findings derived from signal intensity in HBP.

#### Differentiation grade prediction

As OATP expression decreases during hepatocarcinogenesis, HBP signal intensity correlates with tumor differentiation grade. Histological differentiation grade is a critical prognostic factor influencing both treatment selection and patient outcomes. Poorly differentiated HCCs, which typically require more aggressive treatment approaches and have worse prognoses, show more pronounced hypointensity on HBP compared to well- or moderately differentiated tumors [43].

#### Microvascular invasion prediction

Microvascular invasion (MVI) is a critical prognostic factor in HCC, significantly impacting treatment outcomes and overall survival. While gross vascular invasion is usually identifiable through imaging, MVI has been generally considered challenging to detect [44]. However, gadoxetic acid-enhanced MRI has emerged as a valuable preoperative tool for predicting MVI.

**Fig. 14:**
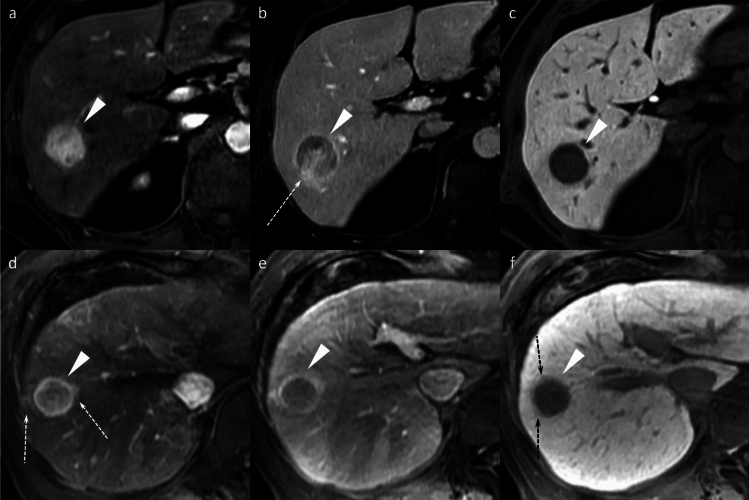
Hepatocellular carcinomas (HCC) in a 60-year-old man and a 74-year-old man. Both HCCs (arrowheads) exhibit typical imaging features, including non-rim arterial phase hyperenhancement (APHE) (**a**, **d**), portal venous washout (**b**, **e**), and marked hypointensity on the hepatobiliary phase image (**c**, **f**), leading to a radiologic diagnosis of HCC. Additional findings such as capsular disruption (**b**; arrow), peritumoral arterial hyperenhancement (**d**; arrows), and peritumoral hypointensity on the hepatobiliary phase image (**f**; arrows) suggest the possibility of microvascular invasion. Histopathological examination after surgical resection confirmed microvascular invasion in both cases

Several imaging features on gadoxetic acid-enhanced MRI are associated with a high specificity for MVI prediction. These include peritumoral hypointensity on HBP (Fig. 14), non-smooth tumor margin, multifocality, larger tumor size (> 5 cm), rim arterial enhancement (Fig. 14), peritumoral arterial enhancement (Fig. 16), and hypointensity on T1-weighted images (Fig. 14) [45]. Among these, peritumoral arterial enhancement and peritumoral hypointensity on HBP showed high specificity but low sensitivity [46]. In addition, combining HBP imaging features with morphological characteristics such as irregular tumor margins and satellite nodules has been shown to further improve MVI prediction accuracy [47]. Conversely, the presence of intratumoral fat is negatively correlated with MVI (Fig. 3) [48].

Given that MVI is a well-established predictor of early recurrence and poor survival following curative treatments, pretreatment identification of high-risk patients is essential for personalized management strategies. In patients presenting with imaging or clinical features suggestive of MVI, anatomical hepatic resection may be recommended over radiofrequency ablation (RFA) [49, 50], although overall survival after RFA is comparable to that after surgical resection in patients with HCCs < 2 cm in diameter [51, 52]. For HCCs demonstrating peritumoral hypointensity in the HBP, which indicates MVI, a wide hepatic resection margin may be required, as studies have shown that wide resection margins are associated with superior recurrence-free and overall survival compared to narrow margins in HCC with MVI [53]. Moreover, peritumoral hypointensity in the HBP, which indicates MVI presence, is significantly associated with early recurrence following resection [54]. In addition, MVI-suggestive findings including irregular tumor margin and arterial rim enhancement, when combined with lower tumor-to-liver signal intensity ratio on HBP images and lower tumor-to-liver apparent diffusion coefficient ratio, are useful for predicting cytokeratin 19 (CK19)-positive HCC, which demonstrates significantly worse recurrence-free survival after curative resection [55].

In the context of locoregional therapies, particularly RFA, the presence of MVI negatively impacts local tumor control due to a higher propensity for microinvasion extending beyond the ablation margin [56]. Combined approaches, such as TACE with RFA or TACE followed by systemic therapy, are increasingly considered for patients with elevated MVI risk [57, 58].

#### Detection of vessels that encapsulate tumor clusters (VETC)

VETC is a distinctive vascular pattern observed in HCC, characterized by CD34^+^ vessels that surround and encapsulate individual tumor cell clusters. This cobweb-like network structure is readily identifiable via pathological imaging analysis. Unlike conventional vascular invasion, the VETC pattern facilitates invasion-independent tumor cell dissemination, enabling tumor emboli to migrate to distant sites without penetrating the vascular wall [59]. The VETC pattern serves as a critical prognostic indicator in HCC management, demonstrating strong associations with adverse clinical outcomes.

HCCs with the VETC pattern often exhibit characteristic imaging findings, including homogeneous APHE, distinct washout, and clear HBP hypointensity, and peritumoral HBP hypointensity. Tumor size and certain clinical markers may also support diagnosis [60, 61]. One HCC subtype, the macrotrabecular-massive (MTM) subtype, frequently exhibits the VETC phenotype. This subtype is associated with immunosuppressive microenvironments, angiogenesis activation, and poor clinical outcomes [66]. MTM and VETC-positive tumors share overlapping imaging features [62].

Patients with VETC-positive HCCs exhibit significantly higher recurrence rates and reduced overall survival compared to those with non-VETC tumors, regardless of treatment options [63–65]. The presence of VETC pattern reflects aggressive tumor biology and indicates a propensity for early metastatic spread and treatment resistance. Therefore, accurate identification and comprehensive assessment of VETC patterns before initial treatment are crucial for optimal patient management and treatment planning.

#### Characteristics of HBP signal intensity and pretreatment assessment

Beyond the prediction of differentiation grade, HBP signal intensity provides valuable insights into the biological characteristics of HCC before treatment initiation and serves as a predictor of treatment response across multiple therapies.

**Fig. 15:**
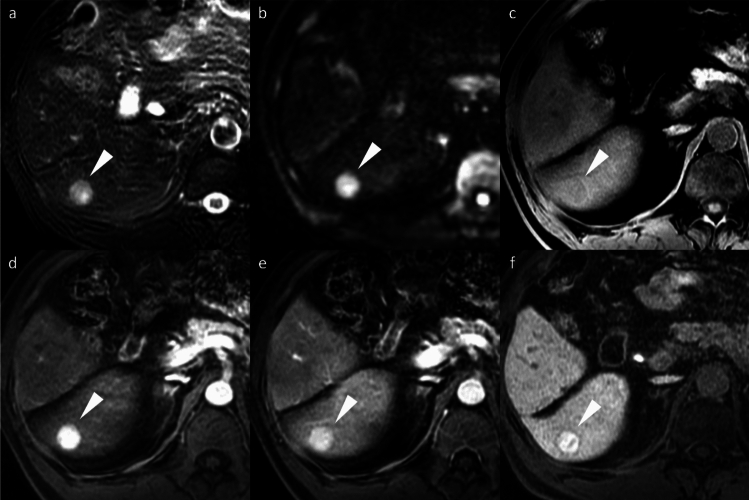
Hyperintense hepatocellular carcinoma in the hepatobiliary phase in a 58-year-old man. A nodule (arrowheads) located in the posterior segment appears clearly hyperintense on fat-suppressed T2-weighted images (**a**) and demonstrates diffusion restriction on diffusion-weighted images (**b**). The nodule displays slight hypointensity on the pre-contrast image (**c**), non-rim arterial phase hyperenhancement (**d**) without portal venous washout (**e**), and clear hyperintensity in the hepatobiliary phase (**f**). The nodule was pathologically proven to be hepatocellular carcinoma following surgical resection

Approximately 10–15% of HCCs exhibit paradoxical hyperintensity in the HBP [43] (Fig. 15), primarily attributed to preserved OATP1B3 expression in tumor cells [66, 67]. This preserved OATP expression is driven by aberrant activation of the Wnt/β-catenin signaling in HCC, typically caused by mutations in the CTNNB1 gene that encodes β-catenin, leading to the cytoplasmic and nuclear accumulation of mutant β-catenin. CTNNB1-mutated HCC is characterized by well-differentiated tumors, with lower rates of microvascular invasion and lower serum alpha-fetoprotein levels [68]. The relationship between HBP signal intensity and treatment response varies significantly across different therapeutic modalities. In conventional transarterial chemoembolization (cTACE), HCC lesions exhibiting iso- to hyperintensity in the HBP show significantly lower local recurrence rates than hypointense lesions [69]. These iso- to hyperintensity lesions have been reported as more sensitive to chemoembolization [70], demonstrating superior treatment outcomes and durability compared to hypointense lesions.

In contrast, HCC with iso- to hyperintensity in HBP has been associated with immune exclusion and resistance to immune checkpoint inhibitors. Among patients with unresectable HCC treated with anti-programmed cell death (PD-1) or PD-L1 monotherapy, those with hyperintense nodules in the HBP had shorter median progression-free survival (PFS) and shorter time to nodule progression than those with hypointense nodules [71]. Similarly, in cases treated with a combination of PD-L1 and multikinase inhibitors, PFS was significantly shorter in patients whose tumors exhibited hyperintensity in the HBP [72]. In contrast, HCCs exhibiting intermediate intensity on HBP could serve as imaging biomarkers for predicting good response to combination therapy with PD-L1 and anti-vascular endothelial growth factor inhibitors [73]. In addition, rim APHE and peritumoral enhancement in arterial phase may also predict favorable response to the same combined therapy.

**Fig. 16:**
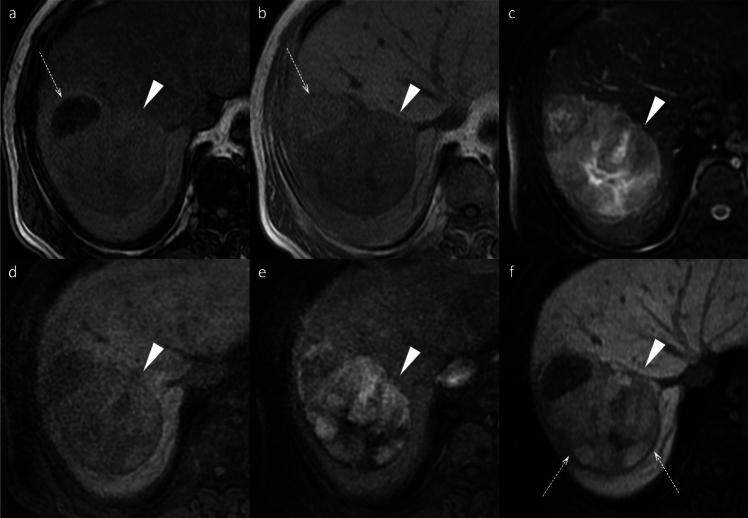
Hepatocellular carcinoma (HCC) showing inhomogeneous hyperintense in the hepatobiliary phase in a 76-year-old man. HCC (arrowheads) includes an area decreasing in signal intensity on the opposed-phase image (**a**) versus the in-phase image (**b**), confirming the presence of intralesional fat (arrows). HCC (arrowheads) also shows slight hyperintensity on the fat-suppressed T2-weighted image (**c**), inhomogeneous hyperintensity on the pre-contrast image (**d**), and non-rim arterial phase hyperenhancement (**e**). In the hepatobiliary phase, HCC (arrowheads) displays inhomogeneous hyperintensity (arrows) (**f**)

Tumor signal heterogeneity in HBP has also been reported to correlate with prognosis during systemic therapy (Fig. 16). Unresectable HCC treated with PD-L1 inhibitor and multikinase inhibitor combination had significantly shorter PFS in cases of HCC showing heterogeneous intensity in the HBP [72]. This heterogeneity likely reflects the presence of various degrees of tumor differentiation and OATP expression within the same lesion, indicating biological diversity that may complicate treatment response prediction.

Furthermore, heterogeneous HBP signal intensity has been reported as a predictor of poor response to drug-eluting bead TACE (DEB-TACE) [74]. Such lesions, composed of areas with varying tumor differentiation and OATP expression, exhibit intratumoral heterogeneity that may result in inconsistent treatment responses within a single tumor. Collectively, these findings highlight the importance of HBP signal characteristics as non-invasive imaging biomarkers for optimizing treatment selection and predicting therapeutic outcomes in HCC management.

### Treatment response assessment

#### Response evaluation criteria of HCC

Traditional criteria such as RECIST and WHO guidelines evaluate HCC treatment response based on tumor size, but these methods have limitations since some therapies cause tumor necrosis without size reduction, and APHE may decrease independently of tumor burden [75, 76]. Chronic liver disease can also mimic progression. To address this, modified RECIST (mRECIST) was developed, focusing on viable tumor tissue and arterial enhancement, and has become the global standard [77].

Both mRECIST and EASL criteria classify responses into four categories: complete response, partial response, stable disease, and progressive disease. mRECIST measures viable lesions in one dimension using arterial phase enhancement, while EASL uses bidimensional measurements including portal venous phase. Meta-analyses show strong correlations with survival outcomes following locoregional and systemic therapies [78]. Current EASL guidelines recommend mRECIST for locoregional therapy assessment and either mRECIST or RECIST for systemic therapy evaluation [78].

The LI-RADS treatment response (LR-TR) system evaluates individual lesions as non-evaluable, non-viable, equivocal, or viable, emphasizing imaging features like washout appearance rather than size. Adding MRI features such as HBP hypointensity, restricted diffusion, and T2 hyperintensity improves viability assessment accuracy within the LR-TR framework [79].

#### Post-locoregional therapy evaluation

**Fig. 17:**
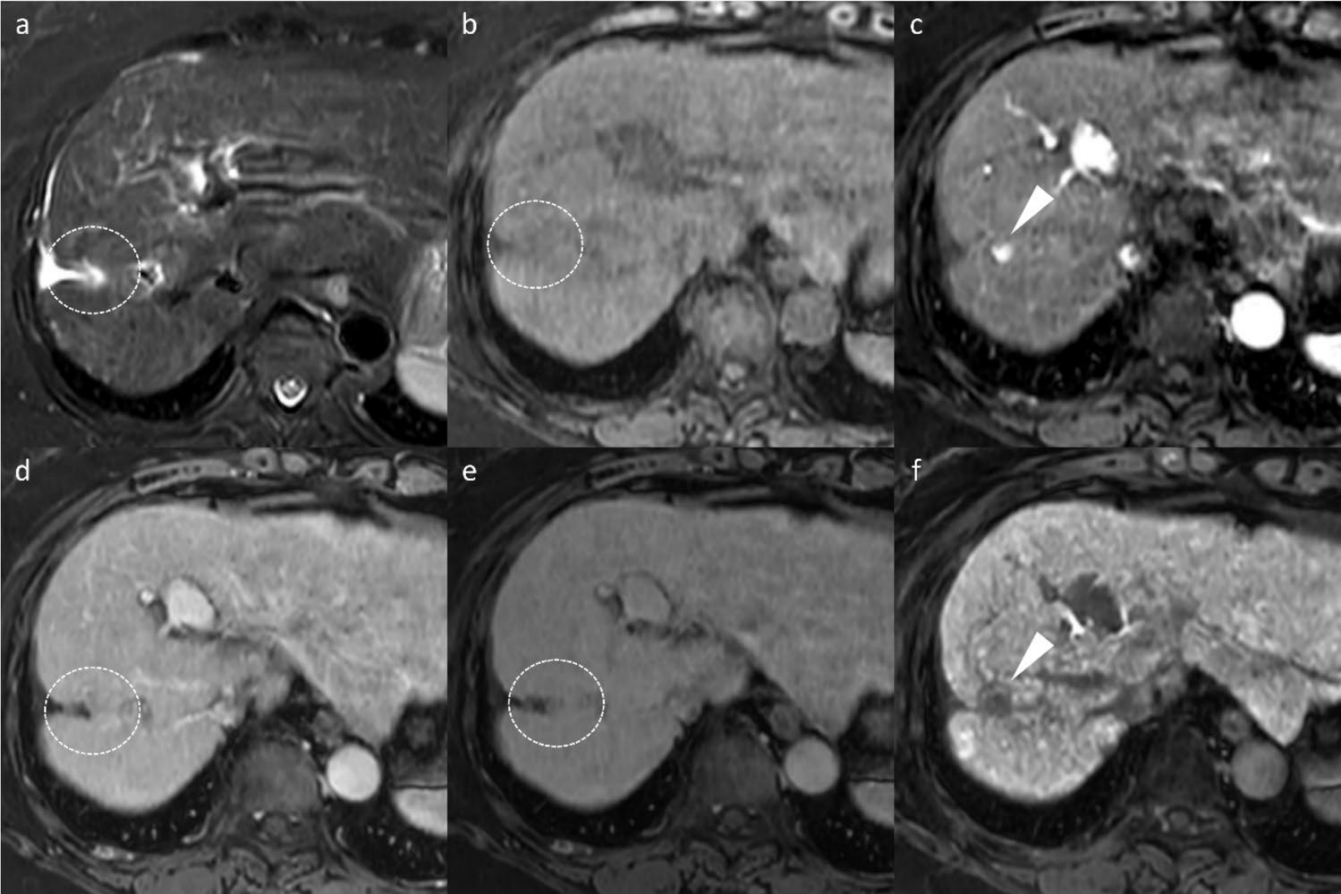
Recurrence of hepatocellular carcinoma after radiofrequency ablation (RFA) in a 75-year-old woman. A small focus of early arterial enhancement (arrowhead) adjacent to the post-RFA ablation zone raises suspicion for tumor recurrence (**c**). The lesion is inconspicuous on fat-suppressed T2-weighted imaging (**a**; dotted circle) and the pre-contrast image (**b**; dotted circle), and lacks definite portal venous washout (**d**; dotted circle) or hypointensity in the transitional phase (**e**; dotted circle). However, the clear hypointensity (arrowhead) observed in the hepatobiliary phase allows for a confident diagnosis of recurrence (**f**)

Gadoxetic acid-enhanced MRI is considered superior to other imaging modalities for assessing treatment response following locoregional therapies such as RFA, TACE, and microwave ablation. Unlike CT, which is often compromised by lipiodol artifacts, gadoxetic acid MRI allows for reliable evaluation of APHE and washout patterns, maintaining high inter-observer reliability. Following RFA, HBP imaging is particularly effective in identifying residual or recurrent tumors, which typically appear hypointense and may exhibit irregular enhancement, washout, and restricted diffusion. This modality also helps differentiate viable lesions from arterioportal shunts (Fig. 17) [80].

#### Systemic therapy response monitoring

Response evaluation for systemic therapies, including immunotherapies and molecular targeted agents, varies based on the mechanism of action. Immune checkpoint inhibitors such as atezolizumab and bevacizumab can cause significant tumor shrinkage, making RECIST criteria applicable [78, 81]. In contrast, targeted therapies such as sorafenib and lenvatinib often do not produce substantial reductions in tumor size, necessitating evaluation of intratumoral hemodynamics. Techniques such as the Choi criteria and dynamic contrast-enhanced MRI have shown better correlation with survival outcomes, particularly in patients treated with lenvatinib, which rapidly reduces blood perfusion [78].

#### Radiation therapy response monitoring

External beam radiation therapy (EBRT), including stereotactic body radiotherapy (SBRT) and particle therapy, is increasingly employed for unresectable HCC patients, enabling precise dose delivery with reduced normal liver irradiation [82].

**Fig. 18:**
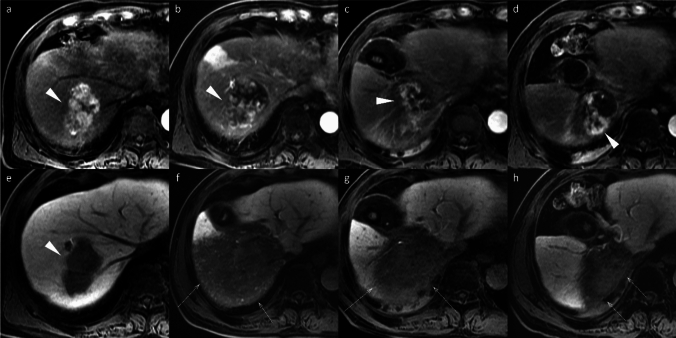
Hepatocellular carcinoma (HCC) in an 80-year-old man treated with proton beam therapy. Prior to treatment, the HCC (arrowheads) demonstrated non-rim arterial phase hyperenhancement (APHE) on the arterial phase image (**a**) and showed a clearly defined hypointense signal on the hepatobiliary phase image (**e**). Following proton beam therapy, the arterial phase images at 3 months (**b**), 6 months (**c**), and 12 months (**d**) revealed gradual tumor shrinkage with a corresponding decrease in APHE, although residual enhancement persisted. On the hepatobiliary phase images at 3 months (**f**), 6 months (**g**), and 12 months (**h**) post-treatment, the irradiated liver parenchyma exhibited decreased gadoxetic acid uptake (arrows), appearing hypointense across the treatment field, with a gradual reduction in volume over time

Gadoxetic acid-enhanced MRI is valuable for assessing EBRT response, though EBRT-specific changes must be recognized. Unlike ablative therapies, radiotherapy may not immediately eliminate tumor vascularity, and residual arterial enhancement may persist for 6–12 months due to radiation-induced inflammation, typically diminishing progressively over time (Fig. 18) [83, 84].

EBRT induces focal liver reaction (FLR) through cellular damage and inflammation. On HBP imaging, FLR appears hypointense, reflecting hepatocyte dysfunction due to OATP1B3 transporter downregulation. These regions can be co-registered with dose distribution maps to estimate threshold dose (TD)—typically 30–40 Gy for normal liver function and 20–30 Gy for compromised hepatic function—enabling individualized treatment planning [85, 86]. Gadoxetic acid-enhanced MRI thus provides objective, quantitative assessment of radiation-induced liver injury beyond traditional clinical and laboratory parameters.

### Limitations and pitfalls of gadoxetic acid

#### Arterial phase timing and transient severe motion

Gadoxetic acid presents several technical challenges that can affect imaging quality. The lower administered dose (0.025 mmol/kg) compared to traditional extracellular agents (0.1 mmol/kg) creates difficulties in achieving optimal arterial phase enhancement, despite the agent’s high relaxivity [6, 87]. In addition, gadoxetic acid-specific transient severe motion artifacts occur in approximately 5–15% of examinations, primarily affecting arterial phase imaging (Fig. 4) [88]. This phenomenon, likely related to transient dyspnea and nausea, can severely compromise diagnostic quality, particularly for detecting APHE lesions.

#### Technical solutions for arterial phase

**Fig. 19:**
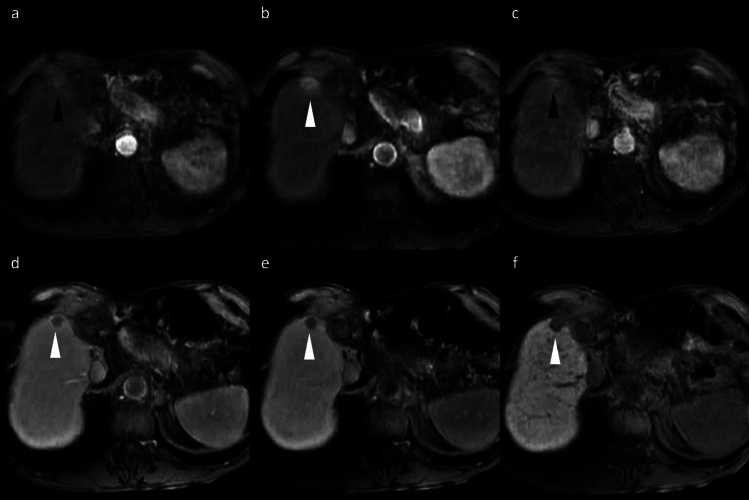
Hepatocellular carcinoma (HCC) in an 80-year-old man with etiology-unknown cirrhosis. Sequential triple arterial phase images are acquired within a single breath hold using gadoxetic acid-enhanced MRI (**a**–**c**). The arterial phase images are compromised by transient severe motion; however, the second arterial phase image captures non-rim arterial hyperenhancement of HCC (**b**; arrowhead). Portal venous washout is seen (**d**; arrowhead). HCC appears hypoenhancing relative to the background liver in the transitional phase (**e**; arrowhead) and shows clear hypointensity in the hepatobiliary phase (**f**; arrowhead)

Multiphasic arterial phase imaging can mitigate these challenges by increasing the likelihood of acquiring at least one image set with optimal scan timing and minimal motion artifacts [7]. Two principal approaches exist. The first is sequential shortened acquisitions, in which the entire k-space for each arterial phase is acquired separately from adjacent phases (Fig. 19) [89]. The second approach involves view sharing, where the central portion of k-space is sampled for each phase, while the peripheral regions are shared across all phases. A recent study using the sequential approach demonstrated that single-breath-hold imaging with three arterial phases reduced motion artifacts and improved overall image quality [89]. Although the clinical utility of view-sharing methods has also been established in prior studies, a notable limitation of multiphasic arterial phase imaging is the variability in APHE subtypes, observed in 28% of cases, which may hinder reliable LI-RADS categorization in at-risk patients. Particularly, the inconsistent expression of rim versus non-rim APHE subtypes complicates accurate imaging diagnosis [90].

Another promising approach is free-breathing dynamic MRI, which reduces the risk of respiratory motion artifacts. This technique often employs a stack-of-stars acquisition approach, combining Cartesian sampling along the z-axis with radial sampling in the XY-plane. Unlike conventional approaches, it allows continuous imaging across the arterial, portal venous, and transitional phases within a single scan, thereby reducing concerns about precise scan timing [91].

#### Considerations for the interpretation of the transitional phase

The transitional phase, representing the interval between the portal venous and HBP, must be interpreted with caution. During this phase, both extracellular and hepatocyte-specific contrast effects coexist, leading to variability in lesion appearance, as the degree of contrast uptake or washout may differ depending on the lesion type, vascularity, and hepatocyte function.

Current CT/MRI LI-RADS guidelines define washout exclusively in the portal venous phase when using gadoxetic acid-enhanced imaging [8]. Washout is not considered a diagnostic imaging feature during the transitional phase, as hepatocyte uptake of the contrast agent begins during this period. Furthermore, enhancing capsule is obscured owing to contrast uptake into the liver parenchyma [92]. These distinctions are critical when comparing gadoxetic acid to extracellular contrast agents.

High-flow hemangiomas might exhibit relatively low signal intensity in the transitional phase due to gadoxetic acid contrast uptake by the surrounding normal liver parenchyma (Fig. 13) [93]. This phenomenon, known as “pseudo-washout,” reflects a relative decrease in signal intensity of the hemangioma compared to the enhancing surrounding liver tissue, rather than true contrast washout from the lesion itself. This pseudo-washout phenomenon of hepatic hemangioma should be distinguished from the true washout seen in HCC during the portal venous phase.

#### Delayed and insufficient gadoxetic acid uptake in the HBP

**Fig. 20:**
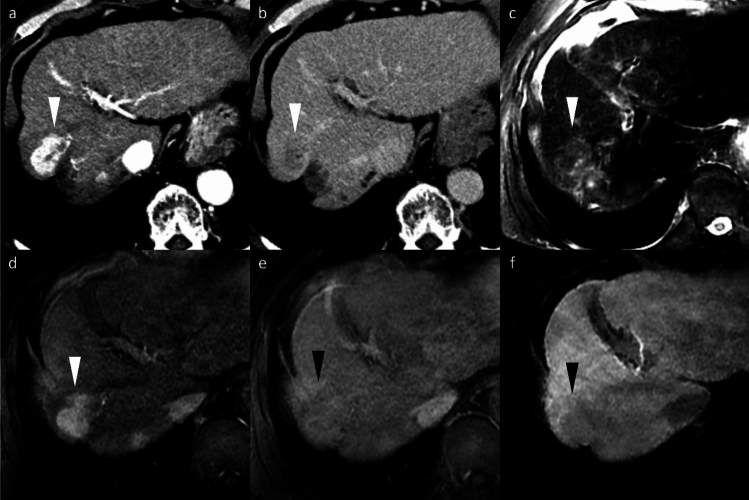
Hepatocellular carcinoma (HCC) in a 71-year-old man with hepatitis C virus-related cirrhosis. HCC (arrowheads) displays non-rim arterial hyperenhancement on the arterial phase CT image (**a**) and portal venous washout (**b**; arrowhead). HCC shows inhomogeneous hyperintensity on the fat-suppressed T2-weighted image (**c**) and demonstrates non-rim arterial hyperenhancement on the arterial phase image (**d**). In contrast, neither portal venous washout (**e**) nor hypointensity on the hepatobiliary phase image (**f**) is clearly observed. Severe liver dysfunction due to advanced cirrhosis decreases gadoxetic acid uptake, thereby reducing the diagnostic contrast between HCC and background liver parenchyma

The diagnostic performance of gadoxetic acid-enhanced MRI in the HBP is contingent on hepatocyte function and integrity. In patients with severe liver dysfunction—including advanced cirrhosis, significant portal hypertension, or acute hepatitis—or concomitant biliary pathology, such as cholangitis, biliary obstruction, or primary sclerosing cholangitis, HBP enhancement may be globally diminished due to decreased gadoxetic acid uptake, thereby reducing the diagnostic contrast between HCC and background liver parenchyma (Fig. 20) [6].

#### T1-weighted imaging limitations

The HBP of gadoxetic acid-enhanced MRI relies on T1-weighted imaging, which can be affected by various parenchymal conditions. Severe iron overload shortens T2* decay in gradient echo sequences, leading to signal loss, while significant hepatic steatosis alters T1 signal intensity depending on echo timing. These changes can reduce liver parenchymal enhancement, potentially masking or mimicking hepatic lesions in HBP, thereby decreasing diagnostic accuracy [94].

#### International guidelines, recommendations, and comparison

Major international hepatology societies have incorporated gadoxetic acid-enhanced MRI into their HCC diagnostic algorithms with varying degrees of emphasis and specific criteria.

LI-RADS has evolved to incorporate gadoxetic acid-enhanced MRI findings, with the 2018 version demonstrating improved diagnostic performance compared to the 2017 version when applied to gadoxetic acid-enhanced examinations [95]. EASL recognizes gadoxetic acid-enhanced MRI as a valuable diagnostic tool, particularly for staging and liver transplantation evaluation. Its updated December 2024 guidelines emphasize individualized imaging approaches based on patient-specific risk factors and clinical context [96]. The American Association for the Study of Liver Diseases (AASLD) emphasizes the utility of gadoxetic acid-enhanced MRI in diagnostically challenging cases where conventional imaging is inconclusive. AASLD maintains a high specificity threshold, with studies reporting a specificity of 97.4% when its criteria are applied to gadoxetic acid-enhanced MRI examinations [96]. In contrast, several Asian guidelines, including those from the Korean Liver Cancer Association–National Cancer Center, the Asian Pacific Association for the Study of the Liver, and the Japan Society of Hepatology, more broadly endorse the use of gadoxetic acid-enhanced MRI. These guidelines generally prioritize higher sensitivity, albeit at the expense of slightly lower specificity [97, 98].

In summary, Eastern guidelines generally emphasize higher sensitivity, whereas Western guidelines prioritize higher specificity. These differences reflect variations in national treatment strategies and resource availability. Incorporating gadoxetic acid-enhanced MRI should be informed by an understanding of these regional distinctions.

## Conclusion

Gadoxetic acid-enhanced MRI offers superior lesion conspicuity and characterization by simultaneously offering both perfusion and functional imaging, making it essential for comprehensive HCC management, including diagnosis, surveillance, and assessment and prediction of treatment response.
